# Characterization of the Clostridioides difficile 630Δerm putative Pro-Pro endopeptidase CD1597

**DOI:** 10.1099/acmi.0.000855.v3

**Published:** 2024-10-08

**Authors:** Bart Claushuis, Arnoud H. de Ru, Peter A. van Veelen, Paul J. Hensbergen, Jeroen Corver

**Affiliations:** 1 Center for Proteomics and Metabolomics, Leiden University Medical Center, Leiden, 2333 ZA, The Netherlands; 2 Leiden University Center for Infectious Diseases, Leiden University Medical Center, Leiden, 2333 ZA, The Netherlands

**Keywords:** *Clostridioides difficile*, MS, proline–proline endopeptidase, protease, sporulation

## Abstract

*Clostridioides difficile* is the leading cause of antibiotic-associated infections worldwide. Within the host, *C. difficile* can transition from a sessile to a motile state by secreting PPEP-1, which releases the cells from the intestinal epithelium by cleaving adhesion proteins. PPEP-1 belongs to the group of Pro-Pro endopeptidases (PPEPs), which are characterized by their unique ability to cleave proline–proline bonds. Interestingly, another putative member of this group, CD1597, is present in *C. difficile*. Although it possesses a domain similar to other PPEPs, CD1597 displays several distinct features that suggest a markedly different role for this protein. We investigated the proteolytic activity of CD1597 by testing various potential substrates. In addition, we investigated the effect of the absence of CD1597 by generating an insertional mutant of the *cd1597* gene. Using the *cd1597* mutant, we sought to identify phenotypic changes through a series of *in vitro* experiments and quantitative proteomic analyses. Furthermore, we aimed to study the localization of this protein using a fluorogenic fusion protein. Despite its similarities to PPEP-1, CD1597 did not show proteolytic activity. In addition, the absence of CD1597 caused an increase in various sporulation proteins during the stationary phase, yet we did not observe any alterations in the sporulation frequency of the *cd1597* mutant. Furthermore, a promoter activity assay indicated a very low expression level of *cd1597* in vegetative cells, which was independent of the culture medium and growth stage. The low expression was corroborated by our comprehensive proteomic analysis of the whole cell cultures, which failed to identify CD1597. However, an analysis of purified *C. difficile* spores identified CD1597 as part of the spore proteome. Hence, we predict that the protein is involved in sporulation, although we were unable to define a precise role for CD1597 in *C. difficile.*

## Data Summary

The mass spectrometry proteomic data are available at the ProteomeXchange Consortium via the PRIDE [1] partner repository with the dataset identifier PXD052347.

Additional data can be found in the supplementary materials.

## Introduction

*Clostridioides difficile*, a Gram-positive opportunistic gut pathogen, is recognized as a leading cause of healthcare-associated infections worldwide [2,4]. A *C. difficile* infection (CDI) manifests primarily as antibiotic-associated diarrhoea, but symptoms range from mild, self-limiting disease to severe and life-threatening pseudomembranous colitis and toxic megacolon [5]. The symptoms of CDI are attributed to the production of potent exotoxins, namely, toxin A (TcdA) and toxin B (TcdB), which disrupt the intestinal epithelial integrity [6]. As an obligate anaerobe, *C. difficile* relies on the production of spores for transmission to new hosts via the faecal–oral route [7].

However, movements of *C. difficile* extend beyond the transmission to new hosts, as the bacteria also travel within the host. In the host, *C. difficile* can exist in a sessile state, adhering to the gut epithelium through adhesion proteins, of which CD2831 and CD3246 are two important players [8,9]. Environmental cues, such as nutrient deprivation, can induce a transition to a motile state, characterized by the release from the gut mucosa and the onset of flagellar production [10]. To detach from the gut wall, *C. difficile* secretes the protease PPEP-1, which cleaves the anchoring substrates CD2831 and CD3246 and thereby releases the cell [8,9].

PPEP-1 belongs to the group of Pro-Pro endopeptidases (PPEPs), comprising secreted zinc metalloproteases with the unique ability to cleave proline–proline bonds [8,11]. Beyond *C. difficile*, PPEP homologues have been predicted in several other bacterial species [12]. The second PPEP that was characterized, PPEP-2 from *Paenibacillus alvei*, displays a distinct specificity from PPEP-1, as both proteases cannot hydrolyse each other’s substrate [11]. On the other hand, PPEP-2 also cleaves a bacterial cell surface protein that is likely involved in adhesion [11], indicating a common function for PPEPs, although alternative roles are conceivable [12].

**Fig. 1.**
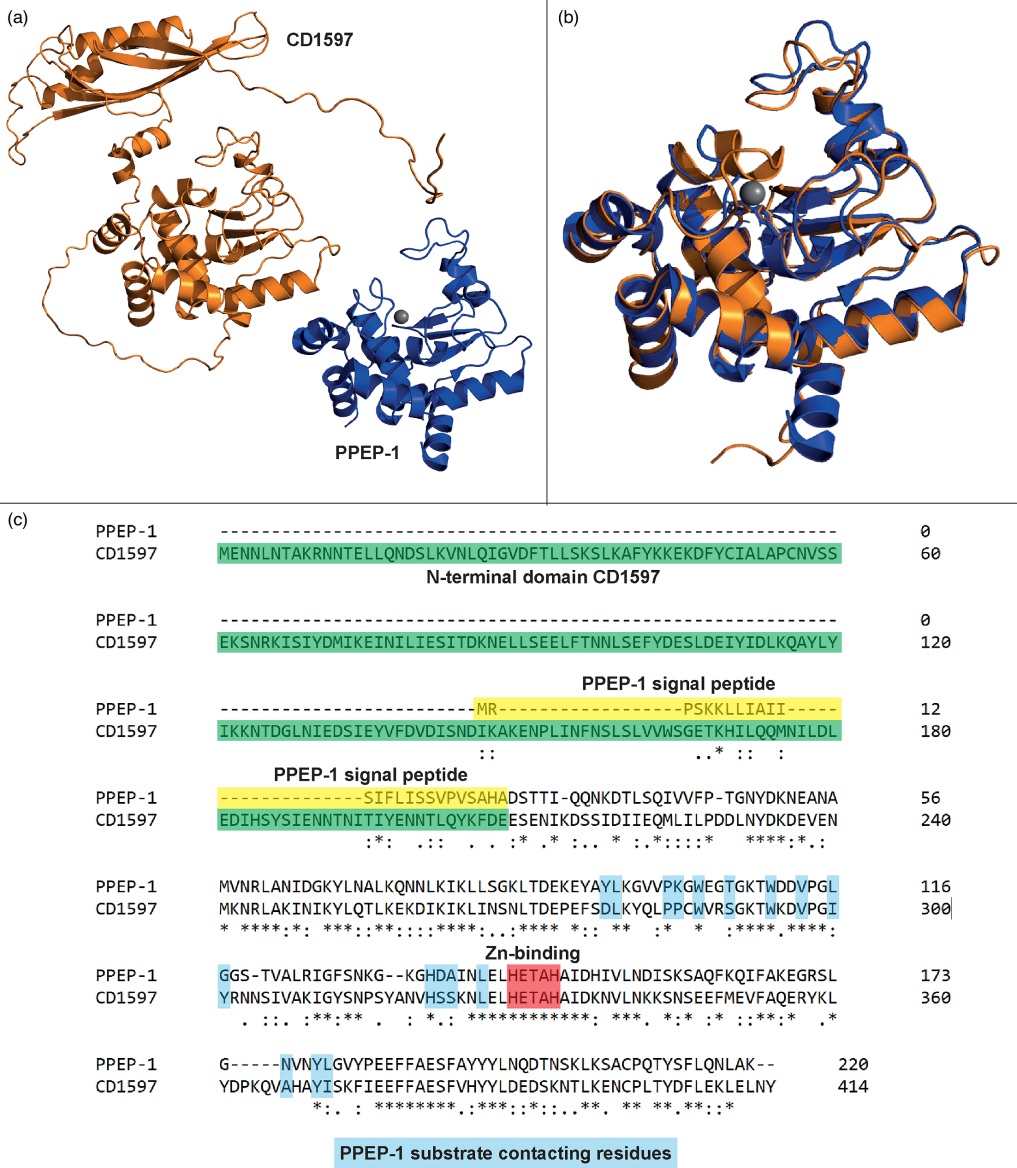
Structural comparison of CD1597 and PPEP-1 from *C. difficile*. (**a**) Predicted structure of CD1597 (AlphaFold, orange) and the crystal structure of PPEP-1 (5A0P, blue). The zinc in PPEP-1 is shown in grey. (**b**) Superimposition of the catalytic domain of CD1597 (AA 211-416, orange) and PPEP-1 (blue). (**c**) Sequence alignment of CD1597 and PPEP-1. Sequence alignment was performed using Clustal Omega [48]. The N-terminal domain of CD1597, the PPEP-1 signal peptide, the HEXXH motif and the substrate contacting residues of PPEP-1 are highlighted. Of note, the first two amino acid residues were removed, as the translation start site is wrongly annotated in the UniProt database (UniProt ID: Q186F3).

Interestingly, a second putative PPEP, CD1597 (UniProt ID: Q186F3), was identified in *C. difficile*. This homologue is distinct from other PPEPs in several ways. First, this putative PPEP lacks a signal peptide for secretion and is presumed to function intracellularly, suggesting a markedly different role for this protein. Second, CD1597 possesses an N-terminal domain of unknown function, constituting approximately half of the protein’s structure (Fig. 1a). This domain is predicted to be linked to the PPEP-like domain through an unstructured (flexible) stretch of residues. Although the presumed catalytic C-terminal domain of CD1597 closely resembles that of PPEP-1 (Fig. 1b), several amino acid substitutions and insertions are observed (Fig. 1c). However, the presence of a zinc-binding HEXXH motif in CD1597 (Fig. 1c) indicates metalloprotease activity [13]. Therefore, we hypothesized that CD1597 is a zinc metalloprotease with PPEP-like specificity [14].

In this study, we sought to uncover the function of CD1597 in *C. difficile* and thereby explore the diversity of roles played by PPEPs in bacteria. To test CD1597 for proteolytic activity, we tested recombinant CD1597 with potential substrates. Moreover, we investigated an insertional *cd1597* (locus tag: CD630_15970) gene mutant for an altered phenotype through a series of *in vitro* experiments and quantitative proteomic analyses.

## Methods

### Growth of bacterial strains and culture media

The strains used in this study are listed in Table 1. *Escherichia coli* strains were cultured in LB broth (Sigma) or LB agar plates supplemented with 25 µg ml^−1^ chloramphenicol, 50 µg ml^−1^ ampicillin or 50 µg ml^−1^ kanamycin when required. *C. difficile* was grown in pre-reduced BHIY [37 g l^−1^ brain heart infusion (Oxoid) supplemented with 5 g l^−1^ yeast extract (Sigma)] or on pre-reduced BHIY agar plates [BHIY supplemented with 15 g l^−1^ agar (Alfa Aesar)]. Alternatively, yeast extract tryptone (YT) (8 g l^−1^ tryptone, 5 g l^−1^ yeast and 2.5 g l^−1^ NaCl), *C. difficile* minimal medium (CDMM) [15] or SMC (90 g peptone, 5 g proteose peptone, 1 g (NH4)2SO4, 1.5 g Tris, containing 0.1% l-cysteine) [16] medium was used. *C. difficile* was cultured anaerobically in a Don Whitley VA1000 or A45 workstation (10% CO_2_, 5% H_2_ and 85 % N_2_ atmosphere) at 37 °C. Media were supplemented with 15 µg ml^−1^ thiamphenicol when required.

### Purification of recombinant CD1597 and the CD1597 catalytic domain

PPEP-1 was expressed and purified as previously described [8]. For the expression of CD1597 and its catalytic domain, pET28a vectors containing *E. coli* codon-optimized 6xHis-*cd1597* (pBC027) and 6xHis-*cd1597* (AA 211-416) (pBC029) constructs were ordered from Twist Bioscience. The expression vectors were transformed to *E. coli* strain C43, and the protein expression was induced using 1 mM IPTG for 4 h at 37 °C. Lysates were made as described in the protocol for the preparation of cleared *E. coli* lysates under native conditions as described in the fifth edition of the QIAexpressionist (Qiagen). A 1 ml HisTrap HP column (GE Healthcare) coupled to an ÄKTA Pure FPLC system (GE Healthcare) was loaded with the lysates. The column was washed using wash buffer (50 mM NaH2PO4, 300 mM NaCl and 20 mM imidazole), and 6xHis-CD1597 and 6xHis-CD1597 (AA 211-416) were eluted using a step gradient with elution buffer (50 mM NaH2PO4, 300 mM NaCl and 250 mM imidazole). The buffer was changed to PBS pH 7.4 using an Amicon 4 ml 3 k centrifugal filter. Glycerol was added to a final concentration of 10% before storage at −80 °C.

### BODIPY TR-X casein cleavage assay

Red-fluorescent BODIPY TR-X casein (EnzChek Protease Assay Kit, Molecular Probes) was used to detect cleavage by CD1597, the CD1597 catalytic domain, PPEP-1 and trypsin. A reaction mixture of 200 µl contained the provided reaction buffer (final concentration 1×), 1 µg enzyme and 1 µg BODIPY TR-X casein. Fluorescence was measured every minute for 60 min in an Envision 2105 Multimode Plate Reader using excitation and emission wavelengths of 590 and 619 nm, respectively.

### Peptide cleavage assays

Kinetics of PPEP activity was measured in a time course using fluorescent Fluorescence Resonance Energy Transfer (FRET) quenched peptides. FRET peptides consisted of Lys(Dabcyl)-EXXPPXXD-Glu(EDANS), in which X varied between the various tested peptides. Cleavage of FRET peptides by PPEPs was tested in 150 µl PBS containing 50 mM FRET peptide and 2 µg of CD1597 or 500 ng for PPEP-1. Peptide cleavage was measured using the Envision 2105 Multimode Plate Reader. Fluorescence intensity (excitation 350 nm and emission 510 nm) was measured each minute for 1 h, with 10 flashes per measurement.

### Growth curves of *C. difficile cd1597*::CT

*C. difficile* 630Δ*erm*, *cd1597*::CT and *tcdC*::CT were grown overnight in BHIY, YT or CDMM medium. For the BHIY growth curves, overnight cultures in BHIY with an OD_600_ <0.9 were used to inoculate fresh BHIY to an OD_600_ of 0.05. For the YT and CDMM growth curves, new precultures were prepared by diluting overnight cultures in their respective media to an OD_600_ of 0.05. These fresh precultures were grown for 2 h to ensure cells were in the exponential growth phase and used to inoculate fresh media to an OD_600_ of 0.05. OD_600_ was measured using an Implen OD600 DiluPhotometer at different time points.

### Generation of a *cd1597*::ClosTron mutant

The ClosTron mutagenesis was performed as previously described [17]. In short, the re-targeting primers for ClosTron mutagenesis of *cd1597* were designed using the intron design tool available at http://clostron.com/ and are shown in Table 1. The re-targeted intron was produced by PCR (with primers CD1597-402/403-IBS, CD1597-402/403-EBS1d and CD1597-402/403-EBS2 and EBS universal primer; see Table 2 for sequences), purified from an agarose gel and inserted in pCR2.1 using a TOPO TA Cloning Kit (Invitrogen). The re-targeted intron was excised from pCR2.1 using *Hind*III and *BsrG*I and ligated into the pMTL007 vector backbone [18] that was digested using the same restriction enzymes. The resulting plasmid was transformed to *E. coli* CA434 and subsequently conjugated to *C. difficile* (see *Conjugation of plasmids to C. difficile*). Conjugants were directly streaked on BHIY plates supplemented with 20 µg ml^−1^ lincomycin. Resistant colonies were grown in BHIY, and gDNA was isolated for PCR with primers oJC147 and oJC148 to test for the spliced *ermB* retrotransposition-activated marker (RAM) intron marker. In addition, to test for the correct insertion site, a PCR was performed with primers oBC084 and oBC085 (targeting the predicted insertion site). The insertion of the *ermB* RAM was also confirmed by Sanger sequencing and whole-genome sequencing (WGS). The resulting *cd1597*::CT strain (BC057) was stored at −80 °C.

**Table 1.** Strains used in this study

Strain	Organism	Genotype	Resistance	Reference
DH5α	*E. coli*	DH5α		
CA434	*E. coli*	CA434		
BC001	*C. difficile*	630∆*erm*		
BC040	*C. difficile*	630∆*erm*, P*cd1597-sLuc*^opt^ (pBC025)	Cam	This study
BC044	*E. coli*	DH5α, pBC062	Amp	This study
BC046	*E. coli*	CA434, pBC063	Cam	This study
BC057	*C. difficile*	630∆*erm*, *cd1597*::CT	Erm	This study
BC062	*C. difficile*	630∆*erm*, P*tet-cd1597* (pBC033)	Cam	This study
BC103	*C. difficile*	630∆*erm*, P*tet-cd1597-cfp*^opt^ (pBC053)	Cam	This study
JC178	*C. difficile*	630∆*erm*, P*ppep1-sLuc*^opt^ (pJC084)	Cam	This study
JC284	*C. difficile*	630∆*erm*, *tcdC*::CT	Erm	[24]
JC336	*C. difficile*	630∆*erm*, *spo0A*::CT	Erm	[49]
WKS1734	*C. difficile*	630∆*erm*, P*tet-cfp^opt^* (pHEW91)	Cam	[22]

**Table 2.** Primers used in this study

Name	Sequence (5′ to 3′)
CD1597-402/403-IBS	AAAAAAGCTTATAATTATCCTTAAACATCGAAGACGTGCGCCCAGATAGGGTG
CD1597-402/403-EBS1d	CAGATTGTACAAATGTGGTGATAACAGATAAGTCGAAGACTCTAACTTACCTTTCTTTGT
CD1597-402/403-EBS2	TGAACGCAAGTTTCTAATTTCGGTTATGTTCCGATAGAGGAAAGTGTCT
EBS universal primer	CGAAATTAGAAACTTGCGTTCAGTAAAC
JC147	ACGCGTTATATTGATAAAAATAATAATAGTGGG
JC148	ACGCGTGCGACTCATAGAATTATTTCCTCCCG
BC084	GTGGATTTTCTTTTGCTTTTATATCATTGC
BC085	GATGAGATTTATATAGACTTAAAACAAGCG
BC090	AAAGAGCTCATTTGAATTTTTTAGGGGGAAAATACCATGGAAAACAATTTAAATACAGCT
BC091	AAACTCGAGACTTCCTGAACCAGATCCTGAATAGTTTAGTTCAAGTTTTTCAAGAAAATC
WKS1070	GTCTTGGATGGTTGATGAGTAC
WKS1071	TTCCTAATTTAGCAGCAGCTTC
WKS1387	CAGATGAGGGCAAGCGGATG
WKS1388	CGTCGGTGAGCCAGAGTTTC

### Conjugation of plasmids to *C. difficile*

Conjugation procedures were conducted as described previously [19]. In summary, the desired plasmid was introduced into the * E. coli* strain CA434 by transformation, and transformants were selected on LB plates supplemented with chloramphenicol (20 µg ml^−1^). A single colony was grown overnight in the LB medium with 20 µg ml^−1^ chloramphenicol. Subsequently, a 1-ml culture pellet from the transformed *E. coli* CA434 was transferred into the anaerobic chamber and combined with 200 µl of an overnight culture of *C. difficile*.

Droplets of this mixture were plated on a BHIY yeast plate and incubated for over 6 h at 37 °C under anaerobic conditions. Following incubation, the bacteria were scraped from the plate with anaerobic PBS, and dilutions were plated on BHIY plates containing 15 µg ml^−1^ thiamphenicol and *C. difficile* selective supplement (Oxoid). Colonies were subjected to three consecutive passages on BHIY plates supplemented with thiamphenicol. After the last passage, the species and the presence of the plasmid were confirmed by PCR. Primers oWKS1070/oWKS1071 (targeting *C. difficile gluD*) and oWKS1387/oWKS1388 (targeting *traJ*, located on the plasmid) were used for this purpose.

All plasmids used in this study are mentioned in Table 3, and all primers used in this study are shown in Table 2.

**Table 3.** Plasmids used in this study

Name	Backbone	Insert	Resistance	Purpose	Reference
pCR2.1	–	–	Amp	TOPO TA cloning of re-targeted intron *cd1597* and *cd1597* for CFP fusion	–
pMTL007	–	–	Cam	Provides backbone for *cd1597* intron	[18]
pBC025	pAP24 [26]	P*cd1597-sLuc*^opt^	Cam	Promoter activity assay	This study
pBC027	pET28a	*cd1597* (*E. coli* codon optimized)	Kan	Expression of CD1597 for purification	This study
pBC029	pET28a	*cd1597* (AA 211–416) (*E. coli* codon optimized)	Kan	Expression of the CD1597 catalytic domain for purification	This study
pBC033	pRPF185	P*tet-cd1597*	Cam	Expression of CD1597	This study
pBC044	pCR2.1	*cd1597*	Amp	Amplification of *cd1597* for CFP fusion	This study
pBC053	pRPF185	P*tet-cd1597-cfp*^opt^	Cam	Expression of CD1597-CFP^opt^	This study
pBC062	pCR2.1	Re-targeted intron *cd1597*	Amp	Amplification of *cd1597* intron for further cloning	This study
pBC063	pMTL007	Re-targeted intron *cd1597*	Cam	Mutagenesis of *cd1597*	This study
pJC084	pAP24 [26]	P*ppep1-sLuc*^opt^	Cam	Promoter activity assay	This study
pRD2	pRPF185	P*tet-hupA-cfp*^opt^	Cam	Backbone for CD1597-CFP^opt^	[22]
pHEW91	pRPF185	P*tet-cfp*^opt^	Cam	Expression of CFP^opt^	[22]

### Promoter activity assay

Overnight cultures of the 630Δ*erm* strain carrying no plasmid, a P*cd1597-*sLuc^opt^ construct (BC040) or a P*ppep1-*sLuc^opt^ construct (JC178) were pelleted, washed once in CDMM and resuspended in CDMM before inoculating BHIY, YT or CDMM medium to an OD_600_ of 0.05. Samples were taken at different time points while measuring the OD_600_. Samples were diluted 1 : 100 in BHIY, and 90 µl of the diluted samples was transferred to white, flat bottom 96-well plates. To each well, 20 µl reconstituted Nano-Glo substrate (50-fold diluted Nano-Glo substrate in kit buffer, Promega) was added, and the plate was incubated for 10 min. Afterwards, relative light units (RLUs) were measured in a GloMax Explorer Multimode Microplate Reader (Promega) using standard settings. The RLUs were corrected for biomass by dividing the RLUs by the OD_600_ value of the bacterial culture at the time of sampling.

### Sample preparation for overall comparative proteomics

For the overall comparative proteomic experiment using the whole cell cultures of *C. difficile*, three biological replicates for * C. difficile* strain 630Δ*erm* and the *cd1597*::CT strain were included. For the *tcdC*::CT strain, two biological replicates were used. As we looked at both the mid-logarithmic and stationary phases in a single experiment, this amounted to a total of 16 samples that were used in a TMTpro 16plex experiment. Single colonies of *C. difficile* strains 630Δ*erm*, *cd1597*::CT and *tcdC*::CT were picked and precultured overnight in BHIY. The precultures were used to inoculate fresh BHIY at a starting OD_600_ of 0.05, and the cells were grown to an OD_600_ of 0.8 before harvesting half of the cells. The remaining cells were grown for 22 h in total. Cells were pelleted by centrifugation (6000 ***g***, 10 min, 4 °C). Pellets were resuspended in 10 ml of ice-cold PBS and washed twice (6000 ***g***, 10 min, 4 °C). After the last wash, pellets were resuspended in 5 ml urea lysis buffer (8 M urea, 50 mM Tris-HCl, pH 7.5 and 1× cOmplete protease inhibitor cocktail EDTA free). Resuspended cells were incubated for 20 min on ice, after which they were lysed by sonication (five bursts of 30 s with cooling on ice in between rounds). After lysis, the tubes were centrifuged (15 min, 15 000 ***g***, 4 °C). Supernatants were transferred to the fresh tubes and stored at −20 °C until use.

For each strain, 100 µg protein in 100 µl ST (5% SDS, 0.1 M Tris-HCl pH 7.5) buffer was used as the starting material. Proteins were reduced using 5 mM tris(2-carboxyethyl)phosphine hydrochloride for 30 min, subsequently alkylated with 10 mM iodoacetamide for 30 min and finally quenched with 10 mM DTT for 15 min, all at room temperature (RT). Next, proteins were precipitated by chloroform–methanol precipitation. For this, 400 µl methanol, 100 µl chloroform and 300 µl dH_2_O were added with vortexing in between each step. After centrifugation (21 130 ***g***, 2 min, RT), the pellet was washed twice with 500 µl methanol. The protein pellet was then resuspended in 100 µl of 40 mM HEPES at pH 8.4 containing 4 µg trypsin and incubated overnight at 37 °C. Subsequently, another 4 µg of trypsin was added and incubated for 3 h at 37 °C.

For the overall comparative proteomic experiments using only spores, five biological replicates were used per strain in a TMTpro 15plex experiment. Single colonies of *C. difficile* strains 630Δ*erm*, *cd1597*::CT and *tcdC*::CT were picked and cultured overnight in the SMC medium. From each overnight culture, 400 µl was spread on two large SMC agar plates (Ø 14.5 cm) for confluent growth. Spores were allowed to develop for 7 days. All cell material was resuspended in 3 ml sterile dH_2_O by scraping and transferred to the tubes and pelleted by centrifugation (3220 ***g***, 10 min, 4 °C). Pellets were washed three times with 10 ml dH_2_O and ultimately resuspended in 10 ml dH_2_O and stored for 4 days at 4 °C. Then, the tubes were centrifuged (15 000 ***g***, 10 min, 4 °C), and the supernatant was removed. Pellets were resuspended in 1.5 ml 20% Gastrografin (Bayer). The cell suspension was carefully layered on top of 10 ml 50% Gastrografin, and the tubes were centrifuged (10 000 ***g***, 30 min, RT) to separate the spores from the vegetative cells. The supernatant was removed, and the spore pellets were resuspended in 500 µl dH_2_O. The spores were washed three times in dH_2_O before storage at −20 °C until further use. The spores were resuspended in 50 µl extraction buffer (4% SDS, 10% 2-mercaptoethanol, 1 mM DTT, 125 mM Tris-HCL at pH 6.8 and 10% glycerol), and proteins were extracted by incubation for 10 min in a heat block set to 108 °C. After centrifugation (15 000 ***g***, 2 min, RT), the supernatant was transferred to a new tube. Again, 50 µl extraction buffer was added to the pellet, and the process was repeated, resulting in a total volume of 100 µl extracted spore proteins.

The total spore protein material per strain (<100 µg) was used as the starting material. A chloroform–methanol precipitation was performed, and the resulting protein pellet was resuspended in the urea lysis buffer. Proteins were reduced, alkylated and quenched as described earlier. After chloroform–methanol precipitation, 2 µg LysC was added, and the mixture was incubated for 2 h at 37 °C. Then, 4 µg trypsin was added, and the tubes were incubated overnight at 37 °C before adding another 4 µg of trypsin and incubated for 3 h.

TMT labelling was performed on 10 µg of tryptic peptides using TMTpro 16plex labelling (Thermo Fisher Scientific, lot no. UK292954 for whole cultures, WK334339 for spores) at RT for 1 h. The excess TMT label was quenched with 5% hydroxylamine for 15 min at RT. From each sample, the labelled peptides were mixed and freeze-dried. The peptides were resuspended in 10 mM ammonium bicarbonate pH 8.4 and split up in 12 fractions on an Agilent Eclipse Plus C18 column (2.1×150 mm, 3.5 µM). Half of the labelled peptides (80 µg) were injected. Mobile phase A was 10 mM ammonium bicarbonate (pH 8.4), while mobile phase B was 10 mM ammonium bicarbonate in 80% acetonitrile (pH 8.4). The gradient was as follows: 2% B, 0–5 min; 2–90% B, 5–35 min; 90% B, 35–40 min; 90–2% B, 40–41 min and 2% B, 41–65 min. The 12 collection vials were rotated every 30 s during sample collection. The 12 fractions were lyophilized and stored at −20 °C prior to liquid chromatography coupled tandem mass spectrometry (LC–MS/MS) analysis.

### LC–MS/MS analyses

Peptides were analysed by online C18 nano-HPLC–MS/MS with an UltiMate 3000 nano-gradient HPLC system (Thermo, Bremen, Germany) and an Orbitrap Exploris 480 mass spectrometer (Thermo). The fractions were injected onto a pre-column (300 µm×5 mm, C18 PepMap, 5 µm, 100 A) and eluted using a homemade analytical nano-HPLC column (30 cm ×75 µm; Reprosil-Pur C18-AQ 1.9 µm, 120 A; Dr. Maisch, Ammerbuch, Germany). The gradient ran from 2 to 40% solvent B (20/80/0.1 water/acetonitrile/formic acid, v/v) in 142 min. The nano-HPLC column, which was drawn to a ∼10-µm tip, functioned as the electrospray needle. The Orbitrap Exploris 480 worked in data-dependent MS/MS mode with a cycle time of 3 s and a Higher energy Collision Dissociation (HCD) of 36 V. The MS2 spectra were recorded in the Orbitrap with a quadrupole isolation width of 1.2 m/z. For MS1, the resolution was 120 000 and the scan range was 400–1500, at standard automatic gain control (AGC) target at a maximum fill time of 50 ms. A lock mass correction of m/z=445.12003 was used. Dynamic exclusion of precursors was set to 45 s after *n*=1, with a precursor range of 10 ppm. Charge states 2–5 were included. For MS2, the first mass was 110 Da. The resolution in MS2 was 45 000 at an AGC target of 200% at a maximum fill time of 60 ms.

### LC–MS/MS data analysis

In the post-analysis process, raw data were converted into peak lists using Proteome Discoverer versions 2.4.0.305 (for analysis of whole cell cultures) and 2.5.0.400 (for analysis of spores) (Thermo Electron) and submitted to the UniProt *C. difficile* 630Δ*erm* database (3752 entries) (Taxon ID: 272563) using Mascot v. 2.2.07 (www.matrixscience.com) for peptide identification. Mascot searches were performed with 10 ppm and 0.02-Da deviation for precursor and fragment mass, respectively, and trypsin was selected as enzyme specificity with a maximum of two missed cleavages. The variable modifications included oxidation (M) and acetyl (protein N-term). The static modifications included TMTpro (N-term, K) and carbamidomethyl (C). Peptides with a False Discovery Rate (FDR) <1% based on Percolator [20] were accepted.

### Sporulation frequencies of strains *cd1597*::CT, 630Δ*erm* and *spo0A*::CT

For the strains *cd1597*::CT and 630Δ*erm*, three individual colonies were used to inoculate 10 ml BHIY supplemented with 0.1% taurocholate and 0.2% fructose. As a negative control, a single replicate for the *spo0A*::CT strain was included. From these cultures, a 10× dilution series was made until a dilution of 10^7^ was reached. Cells were grown overnight. Exponential cultures (OD_600_ <0.9) were diluted to OD_600_=0.5 in fresh BHIY. Next, 250 µl of this suspension was plated on 70 :30 sporulation agar plates [21], and cells were grown for 24 h. Approximately one-eighth of the cells were scraped off the plate and resuspended in 5 ml BHIY. A 5-µl sample was pipetted onto a 1% agarose slab for microscopy. Cells were analysed using a Leica DMB6 phase-contrast microscope. Sporulation frequencies were determined by counting the presence of (developing) spores in the *cd1597*::CT (*n*=7963) and 630Δ*erm* (*n*=5185) cells. For the *spo0A*::CT strain, no sporulation was observed.

### Localization and overexpression of CD1597

A plasmid was constructed for the inducible expression of CD1597, fused to cyan fluorescent protein (CFP). For this, *cd1597* was amplified from *C. difficile 630Δerm* gDNA using primers oBC090 and oBC091. The PCR product was inserted in pCR2.1 using a TOPO TA Cloning Kit (Invitrogen). The resulting plasmid (pBC044) was digested using *Xho*I and *Sac*I, and the insert was ligated into the *Xho*I- and *Sac*I-digested pRD2 backbone [22]. The resulting CD1597-CFP^opt^ plasmid (pBC053) was transformed to *E. coli* CA434 before conjugation to *C. difficile* 630Δ*erm*, producing the strain BC103. As controls, a strain for the expression of CFP^opt^ (WKS1734, harbouring pHEW91), only CD1597 (BC062, harbouring pBC033, plasmid ordered from ATUM) and * C. difficile* 630Δ*erm* were used.

For the analysis using phase-contrast and fluorescent microscopy, the strains were precultured overnight in BHIY. Fresh BHIY was inoculated to an OD_600_ of 0.1, and the culture was grown for 4 h before inducing the expression (only for BC103 and WKS1734) with 0, 25 or 200 ng ml^−1^ anhydrotetracycline (ATc). Cells were imaged 4, 24 and 48 h after induction. At each time point, 1 ml culture was taken and centrifuged for 2 min at 6000 ***g***. The supernatant was removed and the cells were resuspended in 100 µl PBS. Five microlitres of each suspension was pipetted onto 1% agarose slabs on microscopy slides. The cells were imaged with a Leica DM6000 DM6B fluorescence microscope (Leica) equipped with a DFC9000 GT sCMOS camera using an HC PLAN APO 100×/1.4 OIL PH3 objective, using the LAS X software (Leica). A Leica filter set for CFP (11 504 163) was used.

## Results

### Purification of recombinant CD1597

To perform *in vitro* assays using CD1597, both the full-length protein and the predicted catalytic domain (AA 211-416) were recombinantly expressed and purified by immobilized metal affinity chromatography His-tag purification (Fig. S1, available in the online Supplementary Material). Although the purified protein samples predominantly contained CD1597, the full-length purified protein included other proteins as observed by the faint smear on the Coomassie-stained gel (Fig. S1). To ascertain whether these co-purified proteins included other proteases, an LC–MS/MS analysis was performed, and raw data were searched against both *E. coli* and *C. difficile* databases (Table S1). Despite the identification of 150 other proteins other than CD1597, all originating from *E. coli*, none of them were annotated as proteolytic enzymes. In addition, a search for the HEXXH motif, characteristic of metalloproteases, did not reveal any other metalloproteases among the uncharacterized proteins. Based on the LC–MS/MS analysis, we concluded that the purified protein was >90% pure (Table S1).

### Investigation of the proteolytic activity of CD1597

**Fig. 2.**
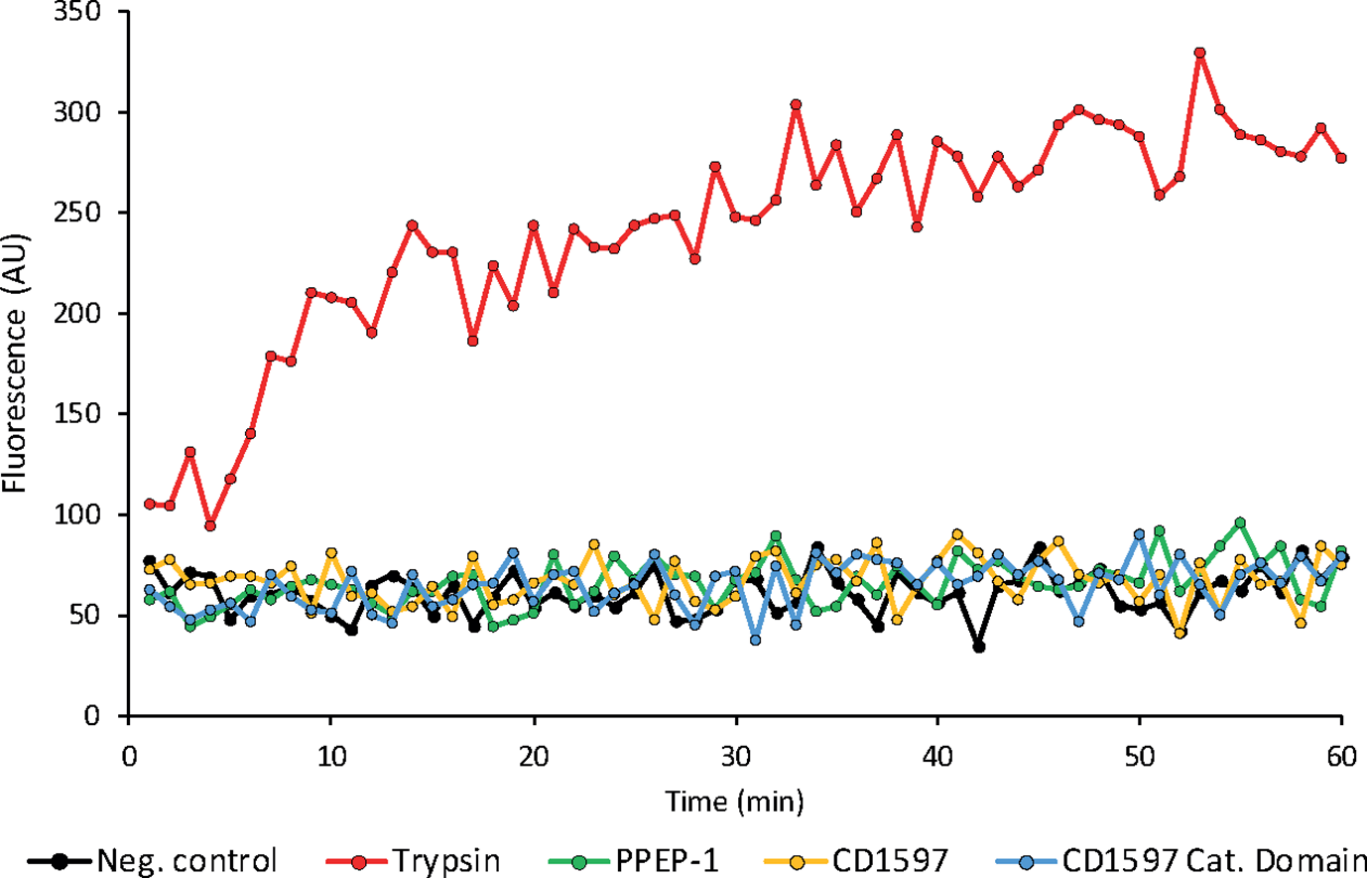
Incubation of BODIPY TR-X casein with CD1597. Proteolysis of the BODIPY TR-X casein substrate relieves quenching of the red fluorescent dye and is observed as an increase in fluorescence. The substrate was incubated with CD1597, the CD1597 catalytic domain, PPEP-1 (PPEP control), trypsin (positive control) and without enzyme (negative control). The data points represent a single technical replicate.

To evaluate the proteolytic activity of CD1597, we initially incubated CD1597 with a BODIPY TR-X casein substrate (Fig. 2). Casein, known for its lack of defined tertiary structure, is considered a generic substrate for many proteases [23]. In addition, one of the constituents, *β*-casein, contains numerous proline residues and also a PPQP sequence, reminiscent of the PPEP-1 cleavage motif [8]. During incubation of BODIPY TR-X casein with CD1597, no increase in fluorescence was observed for both the full-length protein and its catalytic domain, indicating the absence of proteolytic activity towards BODIPY TR-X casein. However, PPEP-1 also did not show any activity towards BODIPY TR-X casein, indicating that casein might not be an appropriate substrate for the highly specific PPEPs.

**Fig. 3.**
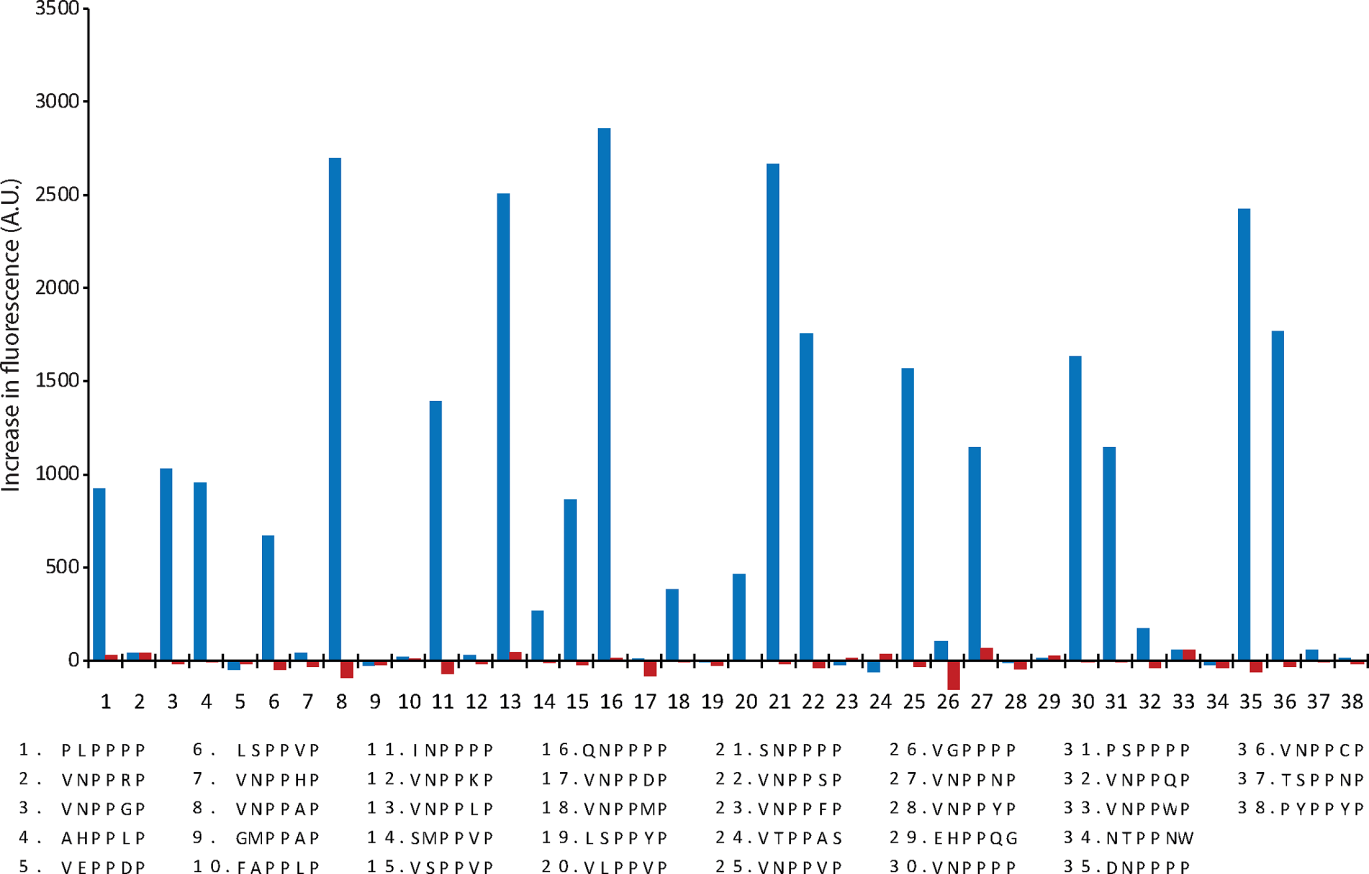
Cleavage of FRET-quenched peptides by CD1597 and PPEP-1. Increase in fluorescent signal after incubation for 1 h with either CD1597 (red) or PPEP-1 (blue) and the synthetic FRET-quenched peptides Lys(Dabcyl)-EXXPPXXD-Glu(EDANS), in which the residues at the X positions vary. For each peptide, the P3-P3′ sequence, containing the fixed Pro-Pro at the P1-P1′, is shown in the legend. The data points represent a single technical replicate.

To assess the activity of CD1597 against Pro-Pro-containing oligopeptides, we incubated CD1597 with a collection of 38 FRET-quenched peptides that were previously used for the characterization of PPEP-1 and PPEP-2 specificity [8,9, 11] (Fig. 3). While PPEP-1 demonstrated proteolytic activity, as evidenced by the increase in fluorescence for multiple peptides, CD1597 exhibited no activity towards any of the peptides. In addition, we tested the catalytic domain using the same set of peptides, but again, no activity was observed (Table S2). These observations suggest that either CD1597 does not possess PPEP activity or it might be inactive towards the specific peptides used.

### Generation of a *cd1597*::ClosTron mutant

**Fig. 4.**
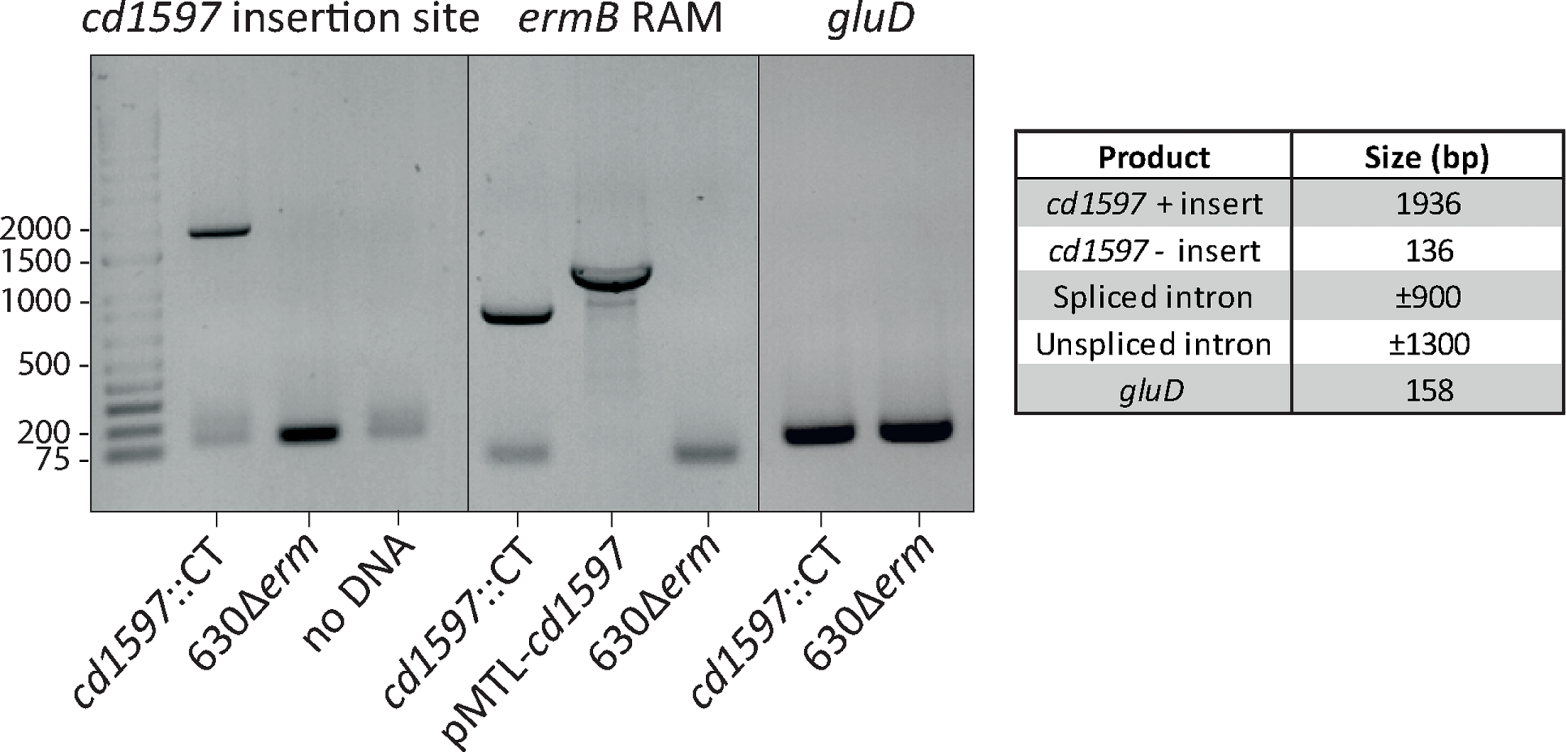
Confirmation of the group II intron insertion in *cd1597* by PCR. A PCR was performed to amplify the region spanning the predicted insertion site (left panel). The faint lower bands for the PCRs with *cd1597*::CT and the no DNA control are most likely primer dimers. A second PCR to amplify the *ermB* RAM intron (middle panel) was performed to discriminate between the spliced and unspliced, i.e. plasmid based, intron. A third PCR confirmed the strain to be *C. difficile* due to the presence of the *gluD* gene (right panel).

Given the absence of detectable proteolytic activity, which could offer insights into potential substrates and hence the functionality of CD1597, alternative approaches involving a CD1597 mutant strain were employed to characterize the protein. For this purpose, a *C. difficile* 630Δ*erm* strain that was deficient in the production of CD1597 was generated by the insertion of a group II intron in the *cd1597* gene using the ClosTron system [18]. The group II intron was designed to insert between bases 402 and 403 of the *cd1597* gene, positioned upstream of the predicted catalytic domain. The correct genotype was confirmed by PCR (Fig. 4) and Sanger sequencing. In addition, WGS verified the insertion of the group II intron at a single locus (data not shown).

### Disruption of *cd1597* does not affect growth rates

**Fig. 5.**
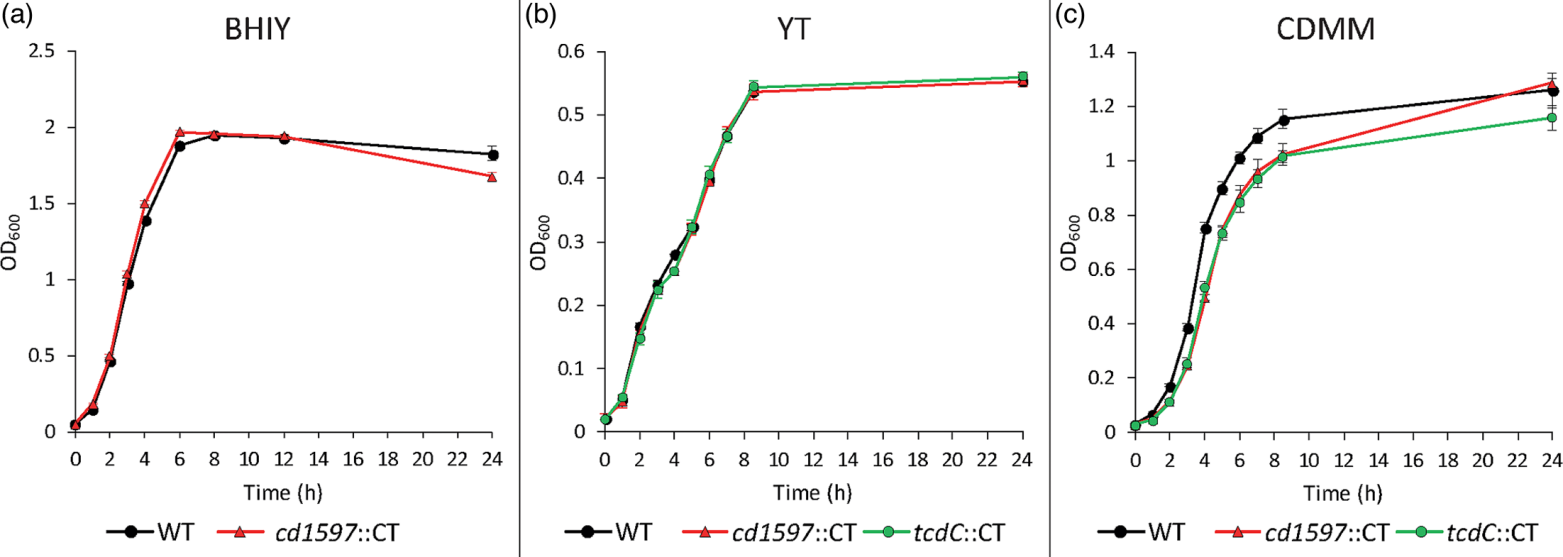
The effect of the disruption of *cd1597* on growth rates in different media. (**a**) Growth curves for the WT (630Δ*erm*) and the *cd1597*::CT strains in BHIY. (**b, c**) Growth curves for the WT, *cd1597*::CT and *tcdC*::CT strains in YT (**b**) and CDMM (**c**). The points in the growth curves are an average of three replicates. Error bars indicate the sd of the OD_600_.

To investigate the effect of the disruption of the *cd1597* gene on the growth rate of *C. difficile*, both the newly generated *cd1597*::CT mutant and the WT 630Δ*erm* strain were grown in the BHIY medium (Fig. 5a). The growth curves in BHIY of both strains were nearly identical, with the exception of the OD_600_ at the 24-h time point, where the OD_600_ was consistently lower for the *cd1597*::CT strain (mean OD_600_ 1.67 vs. 1.83). To assess the potential medium-dependent effects on growth rates, the strains were also grown in YT (which does not contain glucose or cysteine) medium and CDMM [15] (Fig. 5b, c). This time, a *tcdC*::CT strain was included as a control for the ClosTron mutagenesis, although no growth defects were previously observed in the YT medium for this strain [24]. For the growth in the YT medium, no differences were observed between the three strains (Fig. 5b). In CDMM, however, the strains generated by ClosTron mutagenesis did show a reduced growth rate compared to the WT strain (Fig. 5c), although no difference was observed between the *cd1597*::CT and *tcdC*::CT strains during the exponential phase. However, after 24 h of growth, the *cd1597*::CT strain had grown to a similar OD_600_ as the WT strain and therefore differed from the *tcdC*::CT strain. Based on the results in Fig. 5c, it is hard to discern if there is an effect on the growth rate due to the absence of CD1597 or the ClosTron mutagenesis.

**Fig. 6.**
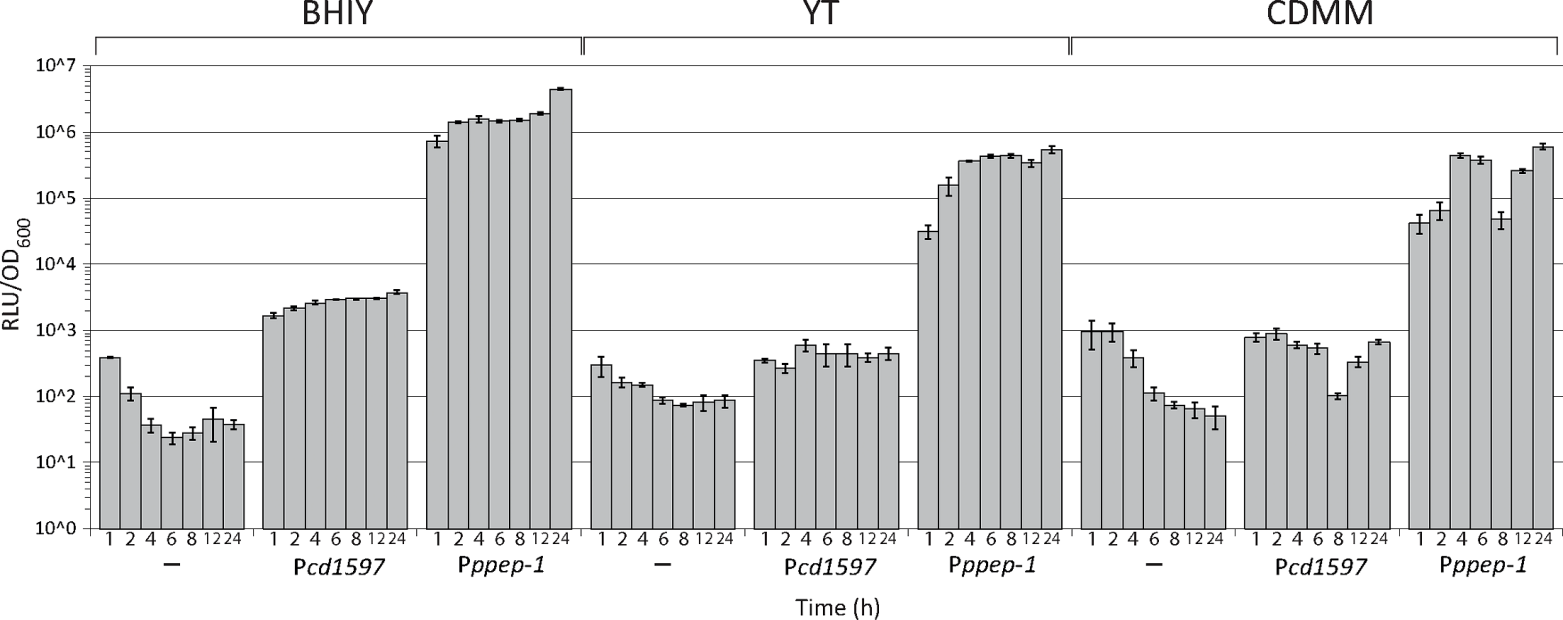
Promoter activity of *cd1597* during growth in different culture media. Strains containing either no plasmid (–), a P*cd1597-sLuc*^opt^ construct or a P*ppep-1-sLuc*^opt^ construct were grown in BHIY, YT and CDMM for 24 h. At different time points, OD_600_ was measured and samples were taken to determine luciferase activity. The relative light units (RLUs) were corrected for the OD_600_ at every time point. The height of the bars shows the average of three biological replicates. The error bars indicate the sd from the mean.

Collectively, there is no clear indication that the disruption of *cd1597* influences the growth rate of *C. difficile*. Possibly, the larger drop in OD_600_ of the mutant in BHIY at 24 h might represent a true growth defect, caused by either an earlier onset of the decline phase or a more rapid decline during this phase. Certain events are most likely to be observed in the most nutrient-rich BHIY, as this allows for the most rapid growth of the bacteria and therefore the earliest onset of the decline phase. Yet, additional experiments were needed to characterize the effect of the absence of CD1597.

### Expression of *cd1597* in different culture media

The analysis of *C. difficile* strain 630 transcriptome data published by Fuchs *et al*. suggested that (1) the expression of *cd1597* is low (e.g. compared to PPEP-1), (2) *cd1597* expression is higher in the YT medium than in BHI and (3) the expression is higher in the late-exponential growth phase (OD_600_=0.9) than stationary phase (3-h post-entry) [25].

To gain more insight into *cd1597* expression, we generated a plasmid containing the *cd1597* promoter (P*cd1597*) upstream of a codon-optimized gene encoding a secreted luciferase reporter molecule (sLuc^opt^) [26]. We monitored the expression of *cd1597* by measuring the luciferase activity during growth and in different culture media, while using *ppep-1* expression as a control (Fig. 6). The luciferase activity, measured by luminescence, was corrected for the OD_600_ at the time of sample collection.

Consistent with the transcriptome data [25], P*cd1597* activity was low compared to P*ppep-1* activity (approximately three orders of magnitude lower) and often only marginally exceeded the negative control. In contrast to the transcriptome data, P*cd1597* activity was higher in the BHIY than YT medium. However, differences in media composition and experimental setup between our study and that of [25] may account for this discrepancy. In CDMM, the luciferase activity was comparable to the negative control during the first 2 h of growth, but P*cd1597*-driven expression of luciferase was observed, as evidenced by the sustained luminescence levels after correction for OD_600_ (as was the case for the negative control). Overall, while PPEP-1 expression levels seemed to increase over time, in line with the model for the regulation of PPEP-1 [10], a similar trend was less evident for CD1597. In BHIY, a slight increase in promoter activity was apparent over time, but this was not mirrored in the other media.

For the negative control lacking a plasmid, the luciferase activity appeared to decrease over time due to the constant background signal being divided by increasing OD_600_. For the CDMM cultures, a sudden drop is observed at 8 h due to low luciferase activity, yet the reason for this is unknown. Nevertheless, as this is observed for both the *cd1597* and *ppep-1* promoters, this is unlikely to represent a biological phenomenon.

Collectively, these findings indicate a consistently low expression of *cd1597* that is not influenced by the growth phase but was highest in the most nutrient-rich BHIY medium.

### Effects of the mutation of *cd1597* on protein levels in *C. difficile* 630Δ*erm*

**Fig. 7.**
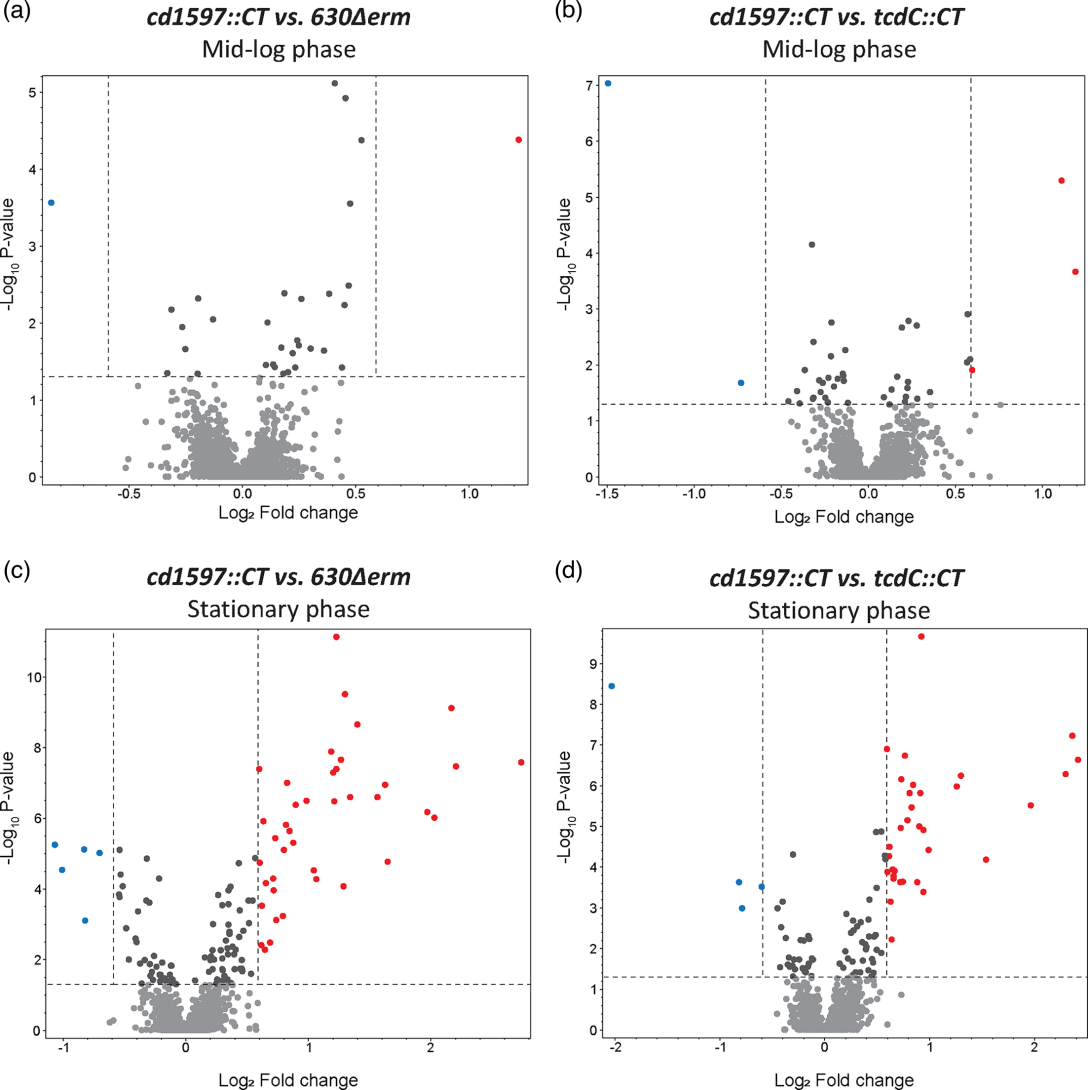
Differences in protein expression in the *cd1597*::CT strain. An overall comparative proteomic experiment using TMTpro 16plex labelling was performed with the WT, *cd1597*::CT and *tcdC*::CT strains. Differences in protein levels were displayed in volcano plots. Each data point represents the average of three biological replicates for the WT and *cd1597*::CT strains and two biological replicates for the *tcdC*::CT strain. Only proteins identified with high confidence and ≥2 PSMs were included in the analysis. Proteins that are >1.5-fold decreased in the *cd1597*::CT strain are shown in blue and those >1.5 increased in red. (**a**) Differently expressed proteins between the *cd1597*::CT and WT strains during the mid-logarithmic phase. (**b**) Differently expressed proteins between the *cd1597*::CT and *tcdC*::CT strains during the mid-logarithmic phase. (**c**) Differently expressed proteins between the *cd1597*::CT and WT strains during the stationary phase. (**d**) Differently expressed proteins between the *cd1597*::CT and *tcdC*::CT strains during the stationary phase.

As there was no evidence of proteolytic activity, a growth defect or a growth phase-dependent expression of *cd1597*, an MS-based quantitative proteomic approach using TMTpro 16plex labelling was taken to investigate differences in protein levels between the WT and the *cd1597*::CT strains. Again, as a control for the potential effects of ClosTron mutagenesis, the *tcdC*::CT strain was included. Bacterial cultures were grown in BHIY and harvested during both the mid-logarithmic phase (OD_600_=0.8) and stationary phase (22 h). Volcano plots were generated to display the differences in protein expression across these cultures (Fig. 7).

We identified 2267 proteins with high confidence and at least two peptides (from a total of 2521 proteins) (Table S3), which, to the best of our knowledge, represent one of the most comprehensive proteomic analyses of *C. difficile* [27,29]. Among the identified proteins was TcdC, which can only originate from the WT and *cd1597*::CT strains. However, the ratio *tcdC*/WT for this protein was 1.01 and 0.45 for the mid-logarithmic and stationary phases, respectively. The fact that the ratio did not approach zero was indicative of the phenomenon called ratio compression [30], which is caused by the co-fragmentation of other peptides in MS2, thereby resulting in an underestimation of the true differences in protein levels. Therefore, we decided to include proteins that showed >1.5-fold increase or decrease in the *cd1597* mutant in our analysis.

During the mid-logarithmic phase, only a few proteins were differently expressed due to the mutation of *cd1597* (Fig. 7a, b). Of note, the protein with the lowest ratio *cd1597/tcdC* in Fig. 7b (upper left blue dot) was ErmB (the antimicrobial resistance gene introduced by ClosTron mutagenesis), which is for reasons unknown more highly expressed in the *tcdC*::CT strain. However, more differences in protein expression were observed during the stationary phase (Fig. 7c, d), indicating that the mutation of *cd1597* primarily impacts the bacteria during this growth phase.

**Fig. 8.**
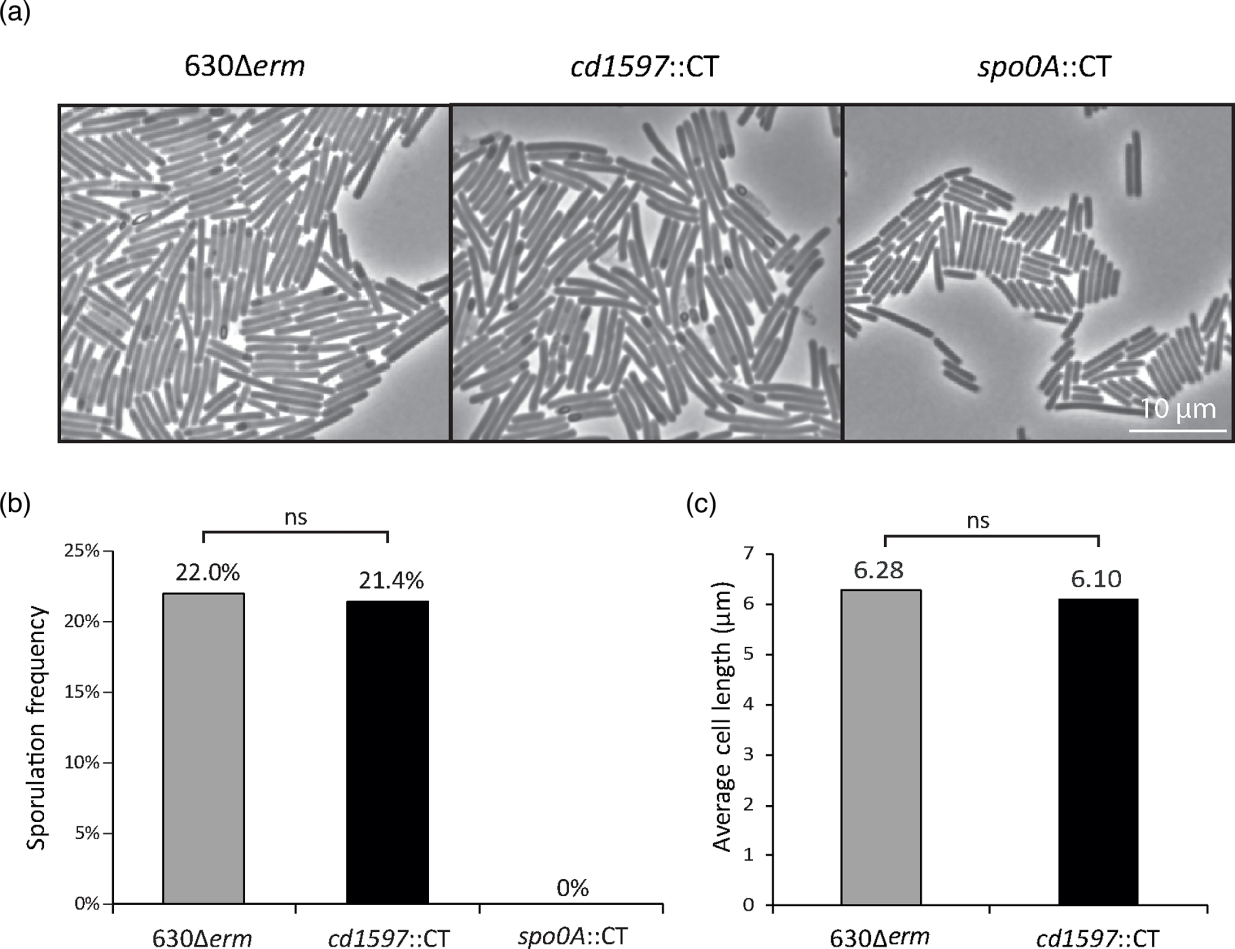
Sporulation frequency and cell length are not affected by the mutation of *cd1597*. (**a**) Phase-contrast micrographs of the WT (630Δ*erm*), *cd1597*::CT and *spo0A*::CT strains. The *spo0A*::CT strain was included as a non-sporulating control. (**b**) Sporulation frequencies were determined by counting the total amount of completed spores and developing spores and dividing this by the total amount of cells (spores+vegetative cells). The difference between the sporulation frequencies of the WT and *cd1597*::CT strains was not significant as determined by a chi-squared test [(*X*^2^ (1, *N*=13 148)=0.641, *P*=0.423]. (**c**) Analysis of cell length of strains 630Δ*erm* and *cd1597*::CT. The length of the cells was determined by analysing the micrographs using Fiji (ImageJ). There was no significant difference in the cell length between the 630Δ*erm* (*M*=6.28, sd=2.02) and *cd1597*::CT (*M*=6.10, sd=2.03) strains as determined by an independent-samples *t*-test; *t*(638)=1.0996, *P*=0.2719.

For the stationary phase, we found 55 proteins to be differently expressed (ratio <0.66 or >1.5) when comparing the *cd1597* mutant to the WT and 45 proteins when comparing the *cd1597* mutant to the *tcdC* mutant, and 39 of these proteins were differently expressed compared to both (Table 4). Several proteins were thus only differently expressed when comparing the *cd1597* mutant to the WT. In addition, of the 39 proteins that were differently expressed in both comparisons, several had a larger difference when comparing the *cd1597* mutant to the WT than to the *tcdC* mutant. This indicated an effect due to the ClosTron mutagenesis rather than the mutation of *cd1597*. Indeed, when comparing our data to similar data of an unrelated strain that was similarly generated using ClosTron mutagenesis [31], we saw a large overlap in differently expressed proteins, corroborating the idea that ClosTron mutagenesis affects protein expression. Therefore, we only included proteins in Table 4 that were differently expressed in the *cd1597* mutant compared to both the WT and the *tcdC* mutant and added a remark if we believed a protein to be differently expressed due to the ClosTron mutagenesis. This remark was based on an integrated analysis that considered whether (1) the proteins showed a similar difference in the expression as in the comparative proteomic data of the unrelated ClosTron mutant [33], (2) the ratio *cd1597*/WT was higher than the ratio *cd1597*/*tcdC* and (3) whether the genes were part of an operon.

**Table 4.** Differently expressed proteins in the cd1597::CT strain during the mid-logarithmic and stationary phases

Increased expression in cd1597::CT during mid-logarithmic phase					
Locus tag	Gene	Description	cd1597/WT	cd1597/tcdC	Remark
CD3275		Putative phosphosugar isomerase	2,33	2,28	

Decreased expression in cd1597::CT during mid-logarithmic phase					
Locus tag	Gene	Description	cd1597/WT	cd1597/tcdC	Remark
–	–	–	–	–	

Increased expression in cd1597::CT during stationary phase					
Locus tag	Gene	Description	cd1597/WT	cd1597/tcdC	Remark
CD1199	spoIIIAH	Stage III sporulation protein AH	6,67	5,35	
CD2688	sspA	Small, acid-soluble spore protein alpha	4,61	5,15	
CD3275		Putative phosphosugar isomerase	4,09	3,92	
CD3249	sspB	Small, acid-soluble spore protein beta	3,92	4,93	
CD1065	cotL	Morphogenetic spore coat protein	3,53	4,39	
CD3567	sipL	Spore coat protein	3,14	2,91	
CD2960	atpI	V-type ATP synthase subunit I	2,96	1,99	Likely ClosTron effect
CD2961		Uncharacterized protein	2,64	1,80	Likely ClosTron effect
CD2629	spoIVA	Stage IV sporulation protein A	2,54	2,40	
CD2656	spoVD	Stage V sporulation protein D (Sporulation-specific penicillin-binding protein)	2,48	2,37	
CD2958	atpE	V-type ATP synthase subunit E	2,47	1,70	Likely ClosTron effect
CD2955	atpB	V-type ATP synthase beta chain	2,41	1,78	Likely ClosTron effect
CD2959	atpK	V-type ATP synthase subunit K	2,34	1,66	Likely ClosTron effect
CD1657	gcvTPA	Multifunctional fusion protein	2,34	1,90	Likely ClosTron effect
CD1658	gcvPB	Probable glycine dehydrogenase (decarboxylating) subunit 2	2,32	1,93	Likely ClosTron effect
CD2954	atpD	V-type ATP synthase subunit D	2,30	1,73	Likely ClosTron effect
CD2956	atpA	V-type ATP synthase alpha chain	2,28	1,76	Likely ClosTron effect
CD0770	spoIIAA	Anti-sigma F factor antagonist	2,09	1,92	
CD2373		Putative CstA-like carbon starvation protein	2,06	1,85	Likely ClosTron effect
CD1935	spoVS	Stage V sporulation protein S	1,98	1,89	
CD2957	atpC	V-type ATP synthase subunit C	1,86	1,53	Likely ClosTron effect
CD0780		Uncharacterized protein	1,84	1,58	Likely ClosTron effect
CD0725		Bifunctional carbon monoxide dehydrogenase/acetyl-CoA synthase, subunit delta	1,80	1,52	Likely ClosTron effect
CD2737		Putative nitrilase/cyanide hydratase and apolipoprotein N-acyltransferase	1,78	1,66	Possibly ClosTron effect
CD0723		Dihydrolipoyl dehydrogenase	1,75	1,57	Likely ClosTron effect
CD0777		Putative membrane protein	1,74	1,56	
CD0772	sigF	RNA polymerase sigma factor	1,70	1,68	
CD0663	tcdA	Toxin A	1,66	1,54	Possibly ClosTron effect
CD2738		Putative cytosine permease	1,65	1,65	Likely ClosTron effect
CD0721		MTHFR_C domain-containing protein	1,64	1,59	Likely ClosTron effect
CD3637		Putative NADPH-dependent FMN reductase	1,64	1,53	
CD0438		Uncharacterized protein	1,58	1,58	
CD0855	oppA	ABC-type transport system, oligopeptide-family extracellular solute-binding protein	1,54	1,55	Likely ClosTron effect
CD0722	metF	Methylenetetrahydrofolate reductase	1,52	1,50	Likely ClosTron effect
CD0779		Peptidase M20 domain-containing protein 2	1,52	1,51	Likely ClosTron effect

Decreased expression in cd1597::CT during stationary phase					
Locus tag	Gene	Description	cd1597/WT	cd1597/tcdC	Remark
CD0268	flgG1	Flagellar basal body protein	0,56	0,66	
CD3192	cwp21	Putative cell surface peptidase, M4 family-cwp20	0,50	0,58	Possibly ClosTron effect
CD0226		Putative lytic transglycosylase	0,48	0,57	

During the mid-logarithmic phase, only a single protein, a putative phosphosugar isomerase, was differently expressed in the *cd1597*::CT strain (Table 4). Interestingly, this protein was also more highly expressed during the stationary phase and therefore the only protein differently expressed during both growth phases.

In the stationary phase, more proteins were differently expressed in the *cd1597*::CT strain, of which many are thought to be the result of the ClosTron mutagenesis itself. However, when considering the proteins that have an increased expression due to the lack of CD1597, a common theme emerges. Most of these proteins are directly linked to sporulation, either by regulating sporulation genes (SigF and SpoIIAA), the development of spores (SpoIIIAH, SpoIVA, SpoVD and SpoVS) or being part of the finished spore (SspA, SspB, CotL and SipL) (Table 4). Of note, the anti-Sigma factor F protein, SpoIIAB, was also highly expressed (ratio *cd1597*/WT=1.55 and *cd1597*/*tcdC*=1.46, Table S3). SpoIIAA and SpoIIAB, located in an operon, regulate SigF-directed transcription of forespore-specific genes involved in the early stages of sporulation [32,33].

Increased protein levels were also observed for the main virulence factors toxin A (Table 4) and, to a lesser extent, toxin B (ratio *cd1597*/WT=1.43 and *cd1597*/*tcdC*=1.34, Table S3). As these toxins are secreted from the cells, no distinction could be made between increased expression or reduced secretion of the proteins based on our data.

The most notable downregulated proteins are CD0226 and FlgG1, which are both part of the flagellar gene cluster (*cd0226-cd0272* [34]). CD0226 is the first gene of an operon, yet a similar downregulation is not observed for the downstream genes (Table S3).

Although we identified a large set of proteins, CD1597 was not identified. This further demonstrated the low expression of this protein, which was also observed in Fig. 6.

Collectively, the most notable difference in protein expression was the increase in sporulation-related proteins during the stationary growth phase.

### Sporulation frequency is not affected in the *cd1597*::CT strains

Due to the elevated levels of several sporulation proteins in the *cd1597*::CT strain, we hypothesized that the sporulation frequency could be affected due to the mutation in *cd1597*. To test this, we conducted microscopy-based assays to evaluate the sporulation frequencies of the WT, *cd1597*::CT and *spo0A*::CT (negative control) strains (Fig. 8a, b). We observed no difference in the sporulation frequency when comparing the *cd1597*::CT strain to the WT. Furthermore, no difference was observed in the average cell length when comparing the WT to the *cd1597*::CT strain (Fig. 8c), and no other phenotypic changes were observed as a result of the mutation of *cd1597*.

### Comparative proteomic analysis of spores of the *cd1597*::CT strain

**Fig. 9.**
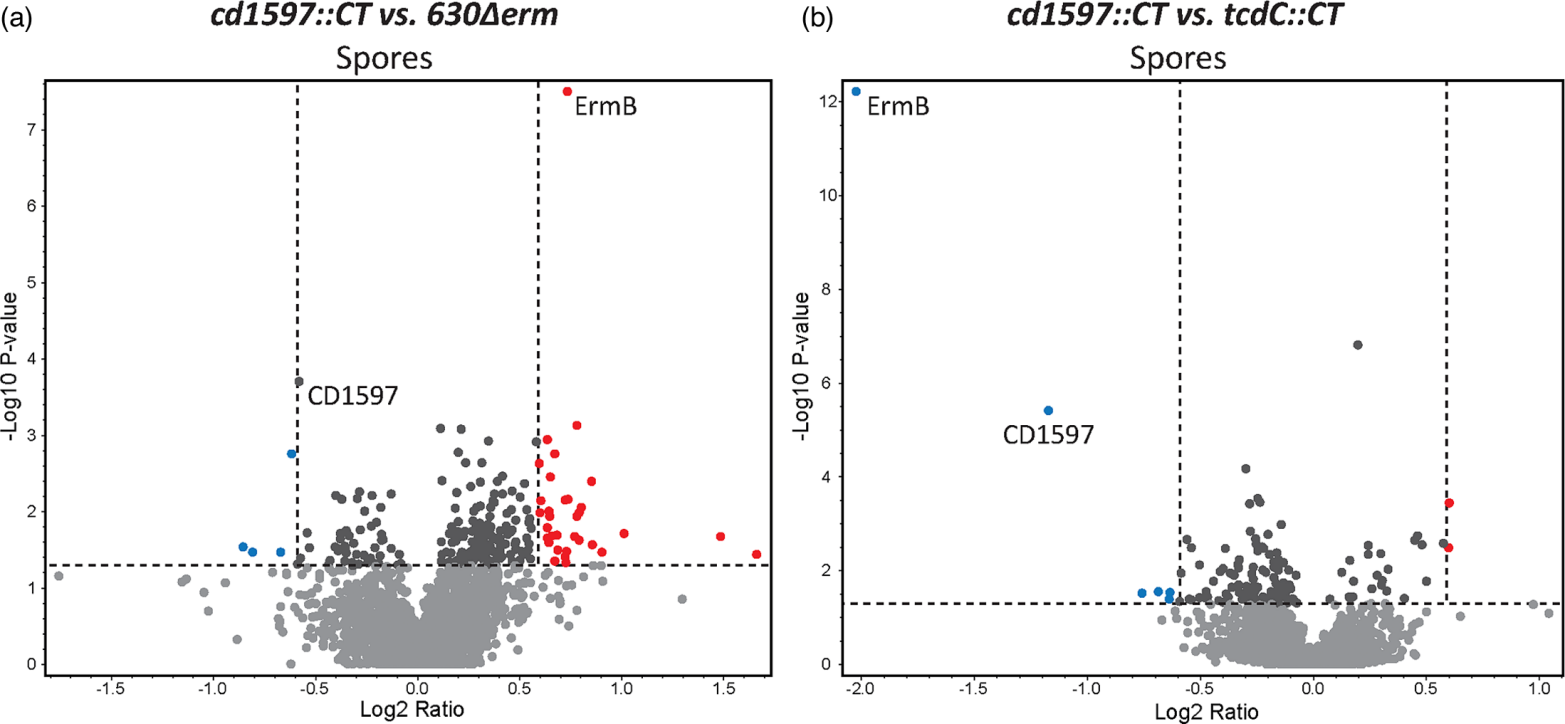
Differences in the spore proteome due to the mutation of *cd1597*. An overall comparative proteomic experiment using TMTpro 16plex labelling was performed on spores of the WT (630Δ*erm*), *cd1597*::CT and *tcdC*::CT strains. Differences in protein levels were displayed in volcano plots. Each data point represents the average of five biological replicates. Only proteins identified with high confidence and ≥2 PSMs were included in the analysis. Proteins that are >1.5-fold decrease in the *cd1597*::CT strain are shown in blue and those >1.5 increase in red. (**a**) Differences in protein levels between the *cd1597*::CT and WT strains. (**b**) Differences in protein levels between the *cd1597*::CT and *tcdC*::CT strains.

Although no differences were observed in the sporulation frequencies, we investigated the spore proteome of the *cd1597*::CT strain in a comparative proteomic analysis of purified spores of *C. difficile* to identify any differences in spore composition (Fig. 9). Again, we included both the WT and the *tcdC*::CT strains as controls.

We identified 2401 proteins with high confidence and at least two peptides (from a total of 2688 proteins) (Table S4). This high number of proteins likely indicated an incomplete separation of the spores from the vegetative cells, but the data also indicated a clear enrichment of spore proteins (e.g. the spore coat proteins). Similar to proteomic analyses of the whole cell cultures (Fig. 7), we found a greater number of proteins to be differently expressed when comparing the *cd1597*::CT strain to the WT strain than when comparing the *tcdC*::CT ClosTron control (Fig. 9). Therefore, we again focused on differently expressed proteins in both comparisons.

First of all, we identified six different peptides of CD1597 in the analysis of *C. difficile* spores (Fig. 9). The fact that CD1597 is exclusively identified after performing LC–MS/MS analysis of spores indicates that this protein is part of the spore proteome rather than the vegetative cell proteome. Although CD1597 could only originate from the WT and *tcdC*::CT samples, we again observed ratio compression, as the ratios for CD1597 do not approach zero (ratio *cd1597*/WT=0.669 and *cd1597*/*tcdC*=0.444).

We looked at proteins that showed >1.5-fold increase or decrease in the *cd1597*::CT strain compared to both the WT and the *tcdC*::CT strains. Three proteins, namely, UxaA’, CD3003 and CD3391, demonstrated higher levels in spores of the cd1597 mutant. However, due to various reasons that included limited peptides/ peptide spectrum match (PSM) identification and high variance, these differences in these protein levels were not considered statistically significant.

Although the mutation of *cd1597* does not greatly influence the spore proteome (apart from the effects of the ClosTron mutagenesis), CD1597 is identified in spores on multiple occasions, suggesting a role for CD1597 in sporogenesis, spore integrity/resistance or germination.

### Localization and overexpression of CD1597

**Fig. 10.**
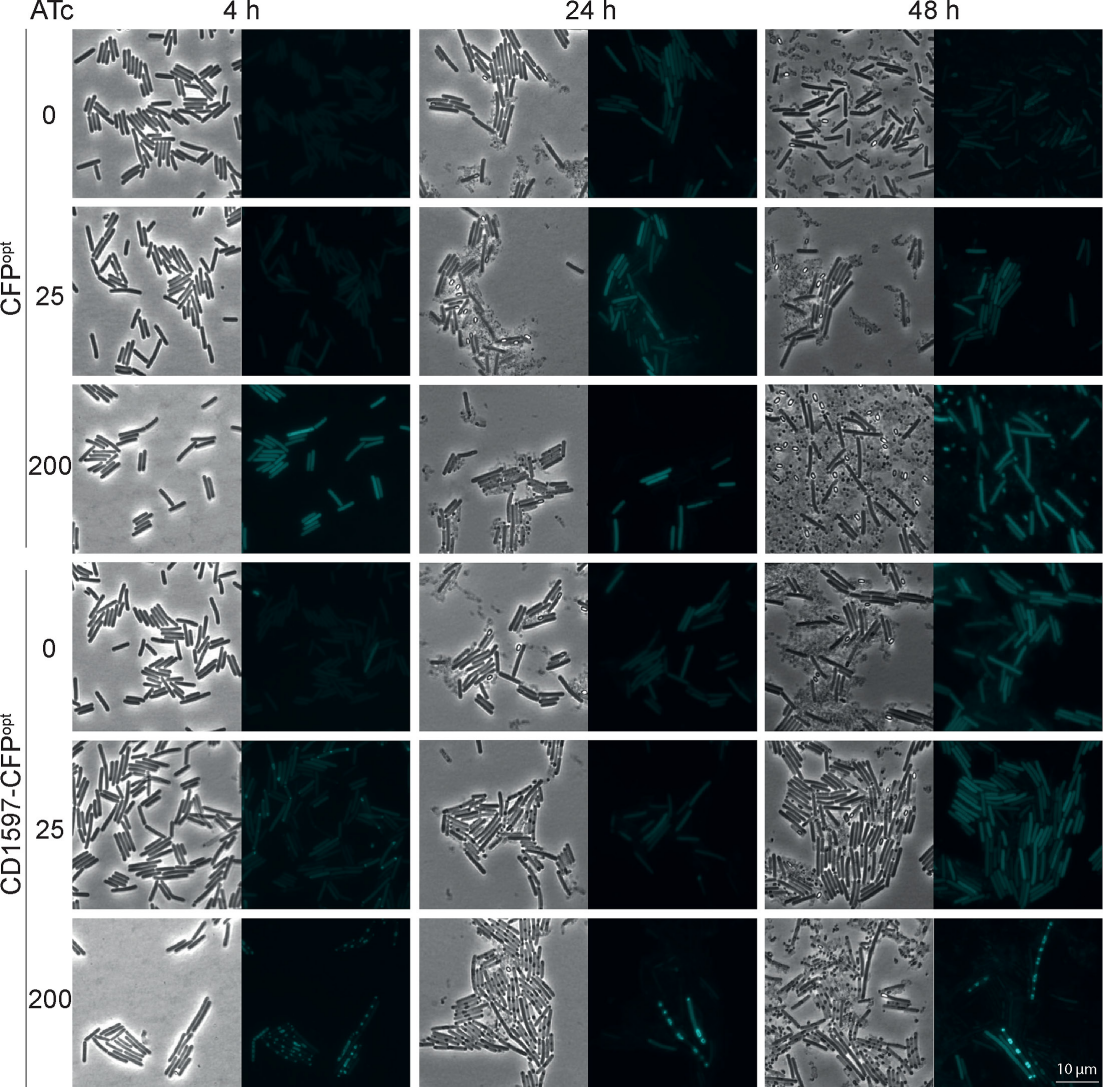
Localization of CD1597-CFP^opt^. *C. difficile* strains containing vectors for the inducible expression of CFP^opt^ or CD1597-CFP^opt^ were grown in BHIY and analysed by phase-contrast and fluorescence microscopy. The expression was induced by adding 25 ng ml^−1^ or 200 ng ml^−1^ ATc. Samples were taken from the cultures after 4, 24 and 48 h.

As CD1597 was identified in spores, we investigated whether CD1597 localizes to the spores during bacterial growth, using cyan fluorescent protein (CFP)-tagged CD1597. For this, we constructed an expression vector containing an inducible *cd1597-cfp^opt^* gene. The localization of the CD1597-CFP^opt^ product was analysed using fluorescence microscopy at different time points and concentrations of the inducer ATc (Fig. 10). As a control, we included a vector that expresses CFP^opt^.

The localization of CFP^opt^ was mostly cytosolic, but at 200 ng ml^−1^ ATc, CFP^opt^ also localized to the spores (Fig. 10). For the CD1597-CFP^opt^, however, we observed a different pattern of localization. After 4 h, CD1597-CFP^opt^ localized to the poles of the cells at 25 ng ml^−1^ ATc, but the higher expression at 200 ng ml^−1^ also showed localization to the midcell, and sometimes, additional CFP signals were observed between the poles and midcell. At 24 and 48 h, the induction of the fusion protein resulted in dark spots in the phase-contrast images, which in most cases did not coincide with a signal in the fluorescent images. Specifically at 200 ng ml^−1^, we also observed the formation of longer cells, suggesting a defect in cell division. In addition, CD1597-CFP^opt^ did not localize to the spores such as CFP^opt^. Together, these results indicate the formation of inclusion bodies that contain the protein aggregates of insoluble CD1597-CFP^opt^. Bacterial inclusion bodies are formed at the cell poles and cause abnormal cell division [35], resulting in a phenotype that is in line with the observations shown in Fig. 10. The inclusion bodies, appearing as dark black spots in the cell, might render CFP non-fluorescent, as, in most cases, these black spots do not produce a fluorescent signal.

**Fig. 11.**
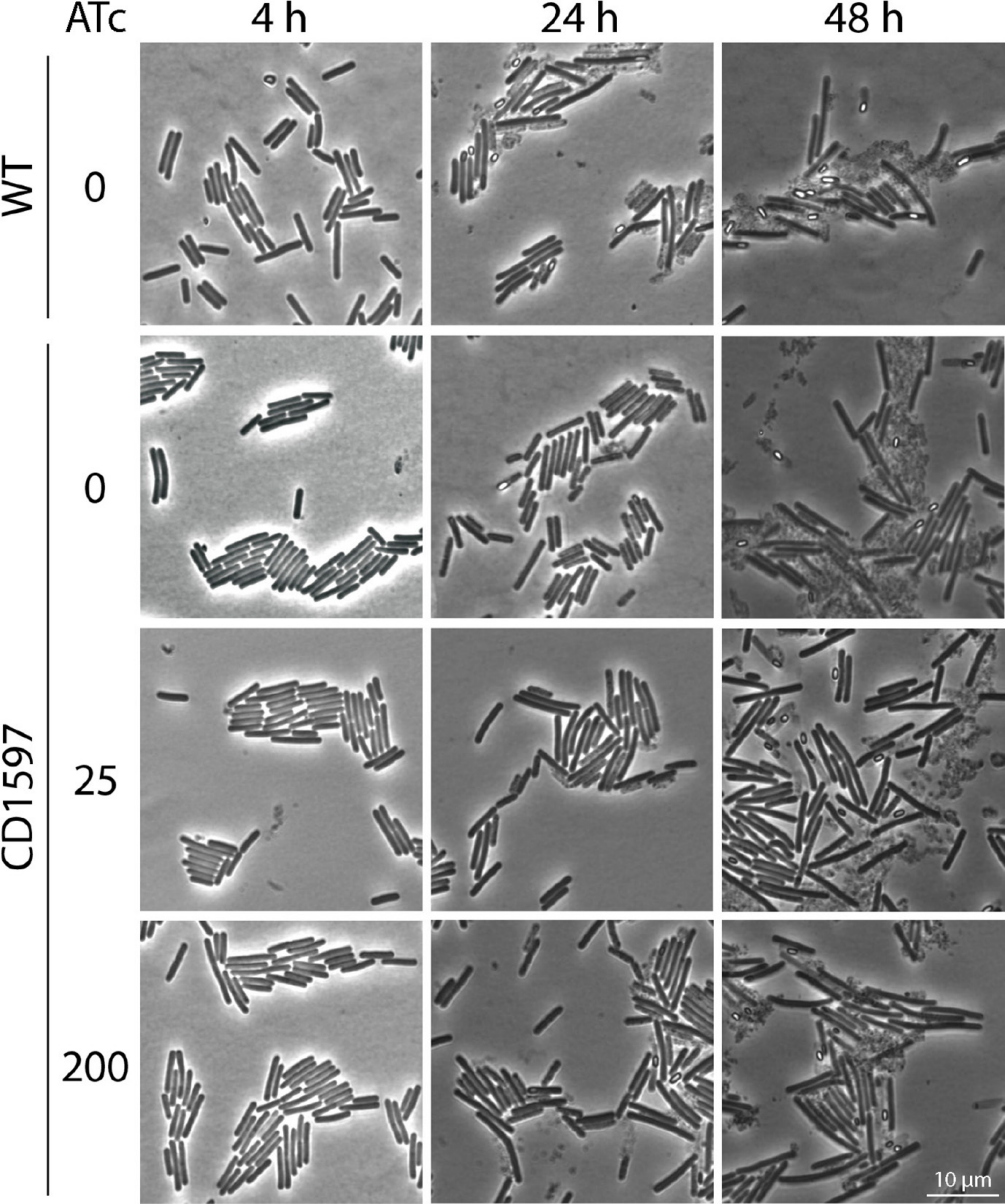
Overexpression of CD1597 does not produce a distinct phenotype. *C. difficile* strains with either no vector or a CD1597 expression construct were grown in BHIY and analysed using phase-contrast microscopy. The expression was induced using 25 or 200 ng ml^−1^ ATc. Samples were taken from the cultures after 4, 24 and 48 h.

To confirm that the observed phenotype resulting from the overexpression of the CD1597-CFP^opt^ fusion protein is due to the formation of inclusion bodies and not due to an effect of CD1597, we analysed the cells that overexpressed CD1597 by microscopy (Fig. 11). The induction of CD1597 did not result in a similar phenotype as observed in Fig. 10. In addition, no difference was observed in either cell length or sporulation between the cultures induced by ATc and the uninduced WT and *cd1597* mutant. Therefore, we conclude that it is not the presence of CD1597 that causes the phenotype observed in Fig. 10, but rather the insolubility of the CD1597-CFP^opt^ fusion protein that leads to the formation of inclusion bodies.

## Discussion

Due to the structural resemblance to other PPEPs, we hypothesized that CD1597 displays PPEP-like proteolytic activity. However, no proteolytic activity was observed in two separate assays. The inability of CD1597 to cleave casein might be unsurprising, as PPEP-1 also lacks this capability. However, also a diverse collection of FRET-quenched peptides containing two consecutive prolines in their core, of which several are cleaved by PPEP-1, were not cleaved by CD1597 and its catalytic domain. Of course, these results do not exclude the possibility that CD1597 is proteolytically active, as we might not have supplied the protein with the correct substrate(s). Assays using peptide libraries that offer a large range of potential substrates might overcome this limitation. However, previous investigations into the activity of CD1597 using a synthetic combinatorial peptide library specifically designed to profile PPEP specificity did not show any proteolytic activity towards Pro-Pro-containing peptides [14]. Alternatively, the substitution and insertion of residues compared to PPEP-1 could render the protein an inactive pseudoprotease, although CD1597 possesses an intact HEXXH domain that suggests metalloprotease activity [36,37].

CD1597 differs from other PPEPs due to the presence of an N-terminal domain. Possibly, the N-terminal domain has an inhibitory effect on the proteolytic activity of CD1597. However, the removal of this domain did not result in activity towards the potential substrates provided in our assays. In addition, we were unable to predict a function for this domain based on bioinformatic analyses that aimed to find proteins with a similar sequence or structure. However, a Foldseek [38] search using the predicted structure of the N-terminal domain of CD1597 (AA 1-211, AlphaFold prediction) provided several proteins from other bacterial species that are predicted to adopt a similar fold. Interestingly, these proteins do not possess additional domains like CD1597 does. However, as no functions have been characterized for these similar folded proteins, we are unable to speculate on the function of the N-terminal domain of CD1597.

Although we observed no obvious growth defect for the *cd1597*::CT strain, the OD_600_ was lower after 24 h than the WT in the BHIY medium, suggesting a larger decline in cell numbers. Around this moment of the growth phase, we harvested our cells for the comparative proteomic experiment and saw an effect of the absence of CD1597. Possibly, differences in protein levels caused by the mutation of *cd1597*, either due to the ClosTron mutagenesis or the lack of CD1597, result in a faster decline of OD_600_ in BHIY after 24 h.

Although we identified a large number of proteins in our analysis of purified spores, which could indicate the presence of vegetative cells in our spore samples, we believe that the identified CD1597 peptides originate from the spores. This belief is strengthened by the fact that we did not identify CD1597 in our LC-MS/MS analysis of the extensively fractionated sample from the vegetative cell cultures. However, we did identify CD1597 peptides in additional label-free LC–MS/MS analyses of *C. difficile* spores (data not shown). In addition, a study by Alves Feliciano *et al*. identified CD1597 peptides in an analysis of the spore coat/exosporium [39], substantiating the idea that CD1597 is part of the spore proteome.

We observed the compression of the ratios (*cd1597*::CT/control) in both proteomic analyses using the OrbiTrap Exploris 480 mass spectrometer and TMTpro labels for quantification, which led to an underestimation of the differences in protein levels. In our experimental setup, this is likely the result of co-eluting ions with similar m/z values. We extensively fractionated our samples on an HPLC system to reduce the co-isolation of ions and also increase protein/peptide identifications. Another method to reduce ratio compression is by performing MS3 fragmentation [40], but this requires alternative instruments. We performed MS3 analysis on an Orbitrap Fusion Lumos instrument using the same spore proteome samples, which slightly improved the ratios for CD1597 but also reduced the number of proteins, peptides and PSMs (data not shown).

ClosTron mutagenesis was used to produce the *cd1597*::CT insertion mutant. An initial comparison of the proteomes of the *cd1597*::CT and the WT strains showed many proteins to be differently expressed. Only after comparing these data to the proteomic data of an unrelated ClosTron mutant [31], we observed an overlap in differently expressed proteins, thereby indicating an effect of the ClosTron mutagenesis itself. Given the frequent use of the ClosTron system in *C. difficile* research, it is challenging to assess the implications of the secondary effects stemming from mutagenesis on the past findings. For example, it has been shown that animals that are challenged with ClosTron mutants have a reduced survival time compared to those challenged with the WT strain [41]. To compare our results with those of others, we searched for quantitative proteomic data from other studies that analysed the full proteome of vegetative cells of both the WT and ClosTron mutants. We identified a single study by Pettit *et al.* [42] that investigated a *spo0A*::CT mutant. The absence of Spo0A, the master regulator of sporulation, has a large and pleiotropic effect on the bacteria and is therefore not suited to compare to our data for investigating the effects of the ClosTron mutagenesis. To study the secondary effects of ClosTron mutagenesis in more detail, studies using an insertional mutation in a non-coding region could identify these secondary effects more precisely, and such a strain could serve as a valuable control strain in experiments using ClosTron mutants.

We observed an increase in both the glucosylating exotoxins TcdA and TcdB from *C. difficile*. However, as these toxins are secreted from the cell, we could not discriminate between an increase in expression and a reduced secretion of these proteins. In our proteomic analysis, we did not identify TcdE, the holin-like protein involved in the secretion of toxins [43], and can therefore not speculate on the amount of secretion based on TcdE levels. In addition, it is unknown whether this increase in cytoplasmic toxins results from the absence of CD1597 or due to the ClosTron mutagenesis. The quantitative proteomic data from the unrelated ClosTron mutant also showed a similar increase in toxin expression, but other studies with ClosTron mutants that exhibited increased toxin expression showed that the toxin levels could be restored by complementation [44,45], indicating that ClosTron mutagenesis is not responsible for elevated toxin levels.

Vector-based complementation of a mutant gene is a powerful tool to prove that the mutated gene is responsible for an observed phenotype. However, in the case of proteomic analyses such as ours, the introduction of the vector for complementation and the selection of this vector using antibiotics had a profound effect on the proteome in our experiments (data not shown). The same was true for vector-based inducible (over)expression constructs, which necessitate an additional molecule for the induction of expression and thereby introduce more variation in the experimental setup. Because of the arguments presented earlier and as we did not observe any phenotypic changes in the *cd1597*::CT strain that could potentially be restored to a WT phenotype, we did not include a complemented strain in our quantitative proteomic experiments.

The only differentially expressed protein during both the exponential and stationary phases was CD3275, a putative phosphosugar isomerase. Phosphosugar isomerases bind phosphosugars and function as an isomerase. However, the substrate and product are unknown, but the *cd3275* gene is located in a predicted phosphotransferase system (PTS) operon for mannose, fructose or sorbose transport and phosphorylation. The other proteins that were more abundant in the *cd1597*::CT strain during the stationary phase were involved in sporulation. Possibly, CD3275 functions as a link between CD1597 and the process of sporulation, and the result of this interplay is only observed during the stationary phase.

Quantitative proteomic analysis of whole cell cultures showed an increase in sporulation proteins during the stationary phase, but no changes were observed in the sporulation frequency of the *cd1597*::CT strain. And, although the effect of the absence of CD1597 was most profound in vegetative cells during the stationary phase, CD1597 was exclusively identified in spores. Furthermore, our proteomic analysis identified only minor, non-significant changes in the spore proteome of the mutant spores. Collectively, our results did not aid us in predicting a role for CD1597. Possibly, CD1597 functions exclusively when the bacteria encounter specific environments or stimuli that were absent in our experiments. Based on the presence of CD1597 in spores, we could expect a role in protecting these spores against environmental challenges or a role in germination. A role in germination is not unthinkable, as other (pseudo)proteases are involved in this process [46,47]. Alternatively, the presence of CD1597 in spores could be a remnant of the sporogenesis, meaning that CD1597 has already fulfilled its role upon completion of the spore. Further investigations into the possibility of proteolytic activity or spore resistance and germination might shed more light on the function of the enigmatic protein CD1597.

## supplementary material

10.1099/acmi.0.000855.v3 Uncited Fig. S1.

10.1099/acmi.0.000855.v3 Uncited Table S1.

10.1099/acmi.0.000855.v3 Uncited Table S2.

10.1099/acmi.0.000855.v3 Uncited Table S3.

10.1099/acmi.0.000855.v3 Uncited Table S4.

## Abbreviations

AGC: automatic gain control
ATc: anhydrotetracycline
BHIY: brain heart infusion
CDI: *Clostridioides difficile* infection
CDMM: *Clostridioides difficile* minimal medium
CFP: cyan fluorescent protein
FDR: false discovery rate
FRET: fluorescence resonance energy transfer
HCD: higher energy collision dissociation
LC-MS/MS: liquid chromatography with tandem mass spectrometry
PPEPs: Pro-Pro endopeptidases
PSM: peptide spectrum match
RAM: retrotransposition-activated marker
RLUs: relative light units
RT: roomtemperature
WGS: whole-genome sequencing
YT: yeast extract tryptone
