# Supplementary material for: Characterization of the Clostridioides difficile 630Δerm putative Pro-Pro endopeptidase CD1597
Source: Access Microbiol. 2024 Oct 8;6(10):000855.v3. doi: 10.1099/acmi.0.000855.v3 (PMC11460543; doi:10.1099/acmi.0.000855.v3)
Supplement: Uncited Fig. S1. [file acmi-6-00855-s001.pdf]

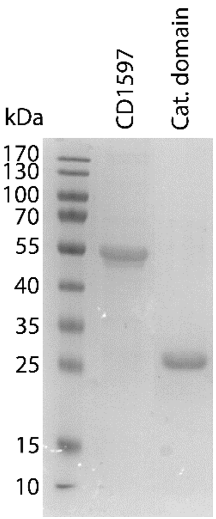

Supplemental Figure S1. SDS-PAGE analysis of purified CD1597. After IMAC His-tag purification, the fractions from the elution peak were pooled, analyzed by SDS-PAGE, and visualized by Coomassie staining. Lanes from left to right: ladder, full-length CD1597, catalytic domain (AA 211-416). The full-length CD1597 and the catalytic domain have a MW of 50.6 kDa and 26.5 kDa, respectively.
